# Supplementary material for: A new investigation of nonalcoholic fatty liver disease: Effects of hypoxia on mitochondrial function and lipid droplet autophagy
Source: PLoS One. 2025 Sep 25;20(9):e0331305. doi: 10.1371/journal.pone.0331305 (PMC12463273; doi:10.1371/journal.pone.0331305)
Supplement: Attachment — Submitted filename: renamed_6715e.docx [file pone.0331305.s001.docx]

**Dear academic editor and** **reviewer**:

Thank you once again for your letter and valuable comments regarding our manuscript titled “A New Investigation of Nonalcoholic Fatty Liver Disease: Effects of Hypoxia on Mitochondrial Function and Lipid Droplet Autophagy” (Manuscript ID: PONE-D-25-15201R2). We sincerely appreciate the constructive feedback you have provided. Your insights have been instrumental in helping us refine the content and analysis of our manuscript, significantly enhancing its quality.

In this revised version, we have diligently addressed all the issues raised by your comments. We have thoroughly reviewed your final remarks and made the necessary corrections, which we believe will meet with your approval. We are truly grateful for your thoughtful and earnest efforts in reviewing our work and hope that these revisions will be satisfactory.

The primary corrections made to the manuscript, along with our detailed responses to your comments, are listed below:

**Journal Requirements:**Please review your reference list to ensure that it is complete and correct. If you have cited papers that have been retracted, please include the rationale for doing so in the manuscript text, or remove these references and replace them with relevant current references. Any changes to the reference list should be mentioned in the rebuttal letter that accompanies your revised manuscript. If you need to cite a retracted article, indicate the article’s retracted status in the References list and also include a citation and full reference for the retraction notice.

**Reply 1:** Thank you very much for your careful review of our reference list and the valuable suggestions you provided. We have followed your request and conducted a comprehensive check of the reference list.

Upon confirmation, all the papers we cited are currently valid, and we have found no instances of retracted literature. Therefore, no deletions or replacements in the reference list are necessary.

We have retained the current reference list in the final version of the manuscript. Additionally, as per the journal’s requirements, we have revised the formatting. Thank you once again for your professional feedback!
